# Histone deacetylase inhibitor, valproic acid, radiosensitizes the C6 glioma cell line *in vitro*

**DOI:** 10.3892/ol.2013.1666

**Published:** 2013-11-07

**Authors:** YONG ZHOU, YING XU, HAN WANG, JUNJIE NIU, HUAYING HOU, YUHUA JIANG

**Affiliations:** 1 Cancer Centre, The Second Hospital of Shandong University, Jinan, Shandong 250033, P.R. China; 2 Department of Radiotherapy, Shandong Jining First People’s Hospital, Jining, Shandong 272011, P.R. China

**Keywords:** histone deacetylase inhibitors, valproic acid, gliomas, C6, radiosensitization, X-ray, apoptosis, *in vitro*

## Abstract

Valproic acid (VPA) is a well-tolerated drug that is used to treat seizure disorders and that has recently been shown to inhibit histone deacetylase. The present study investigated the effects of VPA on the radiosensitization of the rat C6 glioma cell line *in vitro*. To select an appropriate treatment concentration and time, MTT and flow cytometry assays were performed to measure the inhibitory effects of VPA at various concentrations and incubation time-points. The radiosensitizing effect of VPA was determined using clonogenic experiments. VPA- and radiation-induced C6 apoptosis was analyzed using quantitative polymerase chain reaction and western blot analysis. Cell proliferation was significantly inhibited by VPA in a time- and dose-dependent manner (P<0.05). VPA enhanced radiation-induced C6 cell death and there was clear inhibition of clonogenic formation [sensitizer enhancement ratio (SER), 1.30]. This effect was closely associated with the concentration of VPA. VPA treatment decreased the mRNA and protein levels of Bcl-2, whereas increased changes were detected with Bax. At a concentration of 0.5 mmol/l, VPA had a low toxicity and enhanced the radiosensitization of the C6 cells. VPA may radiosensitize glioma cells by inhibiting cellular proliferation and inducing apoptosis by regulating apoptosis-related molecular changes.

## Introduction

Glioblastoma is the most common malignant tumor of the central nervous system in adults. Glioblastoma is an invasive tumor and should be regarded as a disease of the entire brain (1,2). Despite recent advances in surgery, radiotherapy and chemotherapy, there is no effective treatment for glioblastoma, and the progression of the disease is almost always the cause of mortality (3). Radiotherapy is used in the treatment of this disease and shows more potential as concomitant radio-chemotherapy. However, radiotherapy with the highest doses is not possible beyond the tolerance of normal brain tissue. Development of radiation sensitizers that work on brain tumors is therefore required (4,5). Histone deacetylase inhibitors (HDACIs) induce growth arrest, differentiation and/or apoptosis of cancer cells *in vitro* and *in vivo*(6–11). Numerous HDACIs are under investigation in clinical trials, either as monotherapies or in conjunction with other treatments, including chemotherapy, biological therapy or radiation therapy (12). Valproic acid (VPA), a HDACI, is commonly prescribed as an anti-epileptic drug in brain tumor patients due to its effectiveness, oral bioavailability and generally low toxicity profile (13–16). VPA penetrates the blood brain barrier and is chronically administered with minimal toxicity. As with other HDACIs, VPA induces the cytotoxicity and apoptosis of tumor cells and suppresses tumor invasion. Additionally, the action of VPA appears to be specific to glioma cells (17).

Moreover, although VPA has been widely used for the treatment of patients with gliomas, the cellular mechanisms underlying the effects remain unknown. HDACIs enhance the radiation response of various tumor cell types *in vitro* and *in vivo*. The present study examined the effects of VPA and its combination with radiation on the C6 glioma cancer cell line. The effects on glioma cell viability and apoptosis were explored.

## Materials and methods

### Cell line and irradiation

The rat C6 glioma cell line (serial no. 3111C0001CCC000131) was obtained from the Cell Resource Centre (Chinese Academy Of Medical Sciences, Beijing, China) and was cultured in a humidified atmosphere of 95% air and 5% CO_2_ (v/v) at 37°C in Dulbecco’s modified Eagle’s medium [DMEM; 10% fetal bovine serum (FBS), 1% sodium-pyruvate, 1% penicillin/streptomycin, 2 mmol/l L-glutamine, 0.1 mmol/l non-essential amino acids and 1.5 g/l sodium bicarbonate; all from HyClone, Logan, UT, USA]. Experiments were performed with cells that were maintained in the exponential growth phase. VPA was added prior to irradiation (3 Gy/min, 6 MV, X-ray), which was performed in warm culture medium. VPA and MTT (Sigma, St. Louis, MO, USA) were dissolved in phosphate-buffered saline (PBS) to a respective stock concentration of 100 mmol/l and 50 mg/ml, then stored at −20°C. The cells were irradiated using an Elekta Synergy (Stockholm, Sweden) X-ray source. Dosimetry was performed by ion chamber and chemical Fricke dosimetry.

### MTT assay

To determine conditions for VPA treatment, specified numbers (5×10^3^) of cells in single cell suspensions were seeded in individual wells of 96-well plates and incubated for 24 h at 37°C prior to treatment with VPA at indicated concentrations (0.25, 0.5, 1, 2 and 4 mmol/l) for specific time periods (24, 48 and 72 h). Following treatment, MTT solution was added to each well and incubated for 4 h at 37°C prior to removal of the culture medium. Dimethyl sulfoxide (DMSO) was then added and agitated for 30 min at room temperature. Cell viability was determined by measuring the absorbance at 492 nm. Survival data were generated after correcting for cell suppression from VPA alone. Cell suppression ratio (%) = (1 − Ae/Ac) × 100, where Ae is the absorbance of the experimental group and Ac is the absorbance of the control.

### Flow cytometry assay

Cells in the log phase were treated with VPA at indicated concentrations (1×10^6^/ml) or with PBS as a control. Following incubation for an additional 48 h, cells at a specific concentration (1×10^6^/ml) were collected and stained with Annexin V-fluorescein isothiocyanate (FITC) and propidium iodide (PI) for 15 min at 4°C in the dark to identify apoptotic cells by examining nuclear fragmentation and cell membrane permeability under a fluorescence microscope, as per the manufacturer’s instructions (MBL Co. Ltd., Woburn, MA, USA), at the indicated time-points (4°C for 15 min). The extent of apoptosis was quantified using a Becton-Dickinson (Franklin Lakes, NJ, USA) flow cytometer and ModFit LT software (Verity Software House, Topsham, ME, USA).

### Clonogenic assay

To evaluate radiosensitivity, cells in the log phase were plated into individual 25-ml cell culture flasks, which were incubated for 24 h when the cells were attached, but not yet divided. The cells were treated with VPA (0.5 mmol/l) or PBS as a control for 24 h, followed by X-ray irradiation at various doses (0, 2, 4, 6 and 8 Gy), and were then further incubated for 24 h in the presence of the corresponding doses of VPA or not. Prior to plating, the cell culture medium was removed and the cells were washed twice with PBS. Adherent cells were then trypsinized and counted, and five different numbers of cells per dose and substance were seeded in triplicates into tissue culture dishes (Greiner Bio-One, Frickenhausen, Germany) containing fresh, drug-free culture medium without VPA. Colonies were allowed to form over 2–3 weeks. The cell culture medium was then removed and the cells were washed twice with PBS. Colonies were fixed in 100% methanol for 30 min and stained with Giemsa for 15 min. The number of colonies containing ≥50 cells was determined and the number of colonies was normalized to that observed in the unirradiated controls. The sensitizer enhancement ratio (SER) for VPA was calculated as the ratio of the mean inactivation dose of the control divided by the mean inactivation dose of VPA.

### RNA isolation and quantitative polymerase chain reaction (qPCR)

Cells in the log phase were seeded into individual cell culture flasks, which were incubated as aforementioned for 24 h. The cells were treated with VPA (0.5 mmol/l) or PBS as a control for 24 h, followed by 4-Gy radiation or not, and were then incubated for a further 24 h. Prior to isolation, the cell culture medium was removed and the cells were washed twice with PBS. Total RNA was extracted from the cells using TRIzol reagent (Takara Holdings Inc., Otsu, Shiga, Japan) according to the manufacturer’s instructions. cDNA was synthesized from 500 ng total RNA using Takara reverse transcriptase and oligo(dT) primer in a total volume of 10 μl. Reverse transcription was performed at 37°C for 50 min followed by 15 min at 70°C for inactivation. PCR was performed with aliquots of the cDNA samples at an annealing temperature of 60°C with the following specific primers: Bcl-2 (228 bp) forward, 5′-CTGGTGGACAACATC GCTCTG-3′ and reverse, 5′-GGTCTGCTGACCTCACTT GTG-3′; Bax (146 bp) forward, 5′-GCGAGTGTCTCCGGC GAATT-3′ and reverse, 5′-GCCCCAGTTGAAGTTGCC ATCAG-3′; and GAPDH (146 bp) forward, 5′-AAAAGGGTC ATCATCTCCG-3′ and reverse, 5′-AGTCTTCTGAGTGGC AGTGAT-3′. Bax, Bcl-2 and GAPDH amplification reactions were incubated for 15 min at 37°C and then for 30 sec at 95°C, followed by 40 cycles of 95°C for 30 sec, 60°C for 34 sec and 72°C for 20 sec, and a final extension at 72°C for 10 min. Fold changes were calculated in the following manner: Cycle number (Ct) of the target genes was extrapolated from the software analysis program (SDS 1.9; Applied Biosystems, Carlsbad, CA, USA) and then subtracted from the Ct of the input control, and the difference in Ct was known as Delta;Ct. All mean, standard error of the mean and statistical values were calculated as a fold value. To determine the cell radiosensitivity, controls without VPA or X-ray irradiation were also included. Values for enrichment are expressed as a fold change relative to the mean control value.

### Immunoblot analysis

The status of Bax and Bcl-2 was determined by immunoblot analysis. Cytosolic protein fractions and whole-cell lysates for immunoblotting were prepared. For the whole-cell lysates, the cells that were cultivated and treated as aforementioned were lysed in RIPA buffer containing phenylmethanesulfonyl fluoride after washing with cold PBS twice, and then transferred to 1.5-ml microcentrifuge tubes. Five hours after the delivery of irradiation, 1×10^7^ cells were harvested, washed with cold PBS and resuspended in 200 μl modified KCl buffer. Samples were centrifuged at 21,000 × g for 20 min at 4°C. Equal amounts of proteins were separated by 15% SDS-PAGE and transferred onto nitrocellulose membranes according to the manufacturer’s instructions. The non-specific sites on the membrane were blocked at 37°C for 2 h with 5% skimmed milk in Tris-buffered saline supplemented with 0.1% Tween-20 (TBS-T) prior to being washed twice in TBS-T. The membranes were incubated with either rabbit polyclonal anti-Bax, anti-Bcl or goat anti-β-actin antibody diluted in blocking solution overnight at 4°C. The membranes were then washed three times in TBS-T and incubated with the corresponding secondary antibody conjugated with horseradish peroxidase at 1:2,000 dilution in blocking solution for 2.5 h at room temperature. Subsequent to being washed thoroughly three times in TBS-T, the membranes were developed by enhanced chemiluminescence. β-actin was used as a loading control. Images were processed with Image J software (National Institutes of Health, Bethesda, MD, USA) for densitometric quantification. All measurements were performed in triplicate.

### Statistical analysis

For the statistical analyses, SAS software (version 9.1; SAS Institute, Cary, NC, USA) was used. All values are expressed as the mean ± standard deviation. Mean values were compared between three different groups using two-sample t-tests or a one-way analysis of variance. P<0.05 was considered to indicate a statistically significant difference.

## Results

### VPA reduces survival of C6 cells in a dose- and time-dependent manner

VPA inhibited the proliferation of the rat C6 glioma cells in a time- and dose-dependent manner, as determined by MTT analysis (Table I). VPA at 0.25, 0.5, 1, 2 and 4 mmol/l decreased cell viability 24, 48 and 72 h after spreading. The incubation of the cells with 0.25 mmol/l VPA for 24 h resulted in only a modest decrease in cell survival. However, 4 mmol/l VPA reduced cell survival to ~50% compared with the untreated cells.

**Figure 1:**
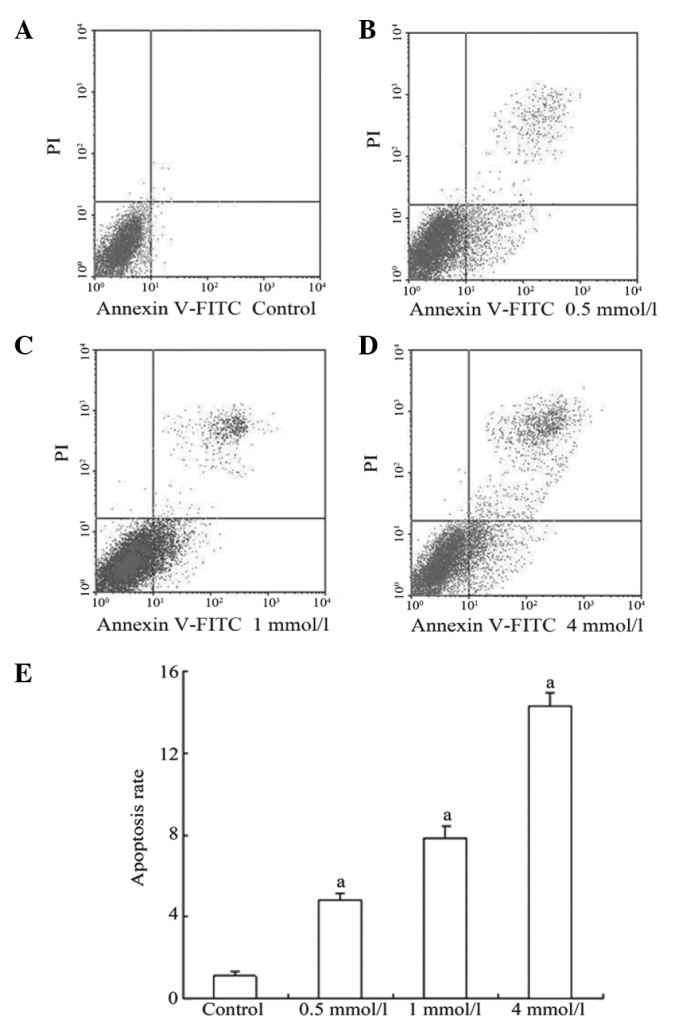
Rate of apoptosis following VPA treatment. Cells were exposed to (A) PBS as a control, or VPA at (B) 0.5 mmol/l, (C) 1 mmol/l or (D) 4 mmol/l. The cells were then collected after 48 h, and apoptosis was assessed by Annexin-V-fluorescein isothiocyanate (FITC) staining and flow cytometric analysis. (E) There was an observed increase in the apoptosis rate in the test groups. Data represent the average of three experiments. Error bars represent one standard deviation. ^a^P<0.05, compared with untreated controls. VPA, valproic acid; PBS, phosphate-buffered saline.

HDACIs are known to induce apoptosis in a variety of cancer cell-lines (6,16). The present study further investigated whether the decreased cell survival in the C6 cells resulted from cell apoptosis. To further quantify cell apoptosis induced by VPA, cell apoptosis was assayed by Annexin V-FITC staining and flow cytometric analysis. As shown in Fig. 1, 0.5 mmol/l VPA treatment increased the apoptotic rate from 1.133±0.166 to 4.768±0.348%. This rate was further increased at 4 mmol/l.

Therefore, a final concentration of 0.5 mmol/l VPA for 48 h was determined to be the optimal condition to treat the cells.

### VPA enhances the radiosensitization of C6 cells in a dose-dependent manner

**Figure 2:**
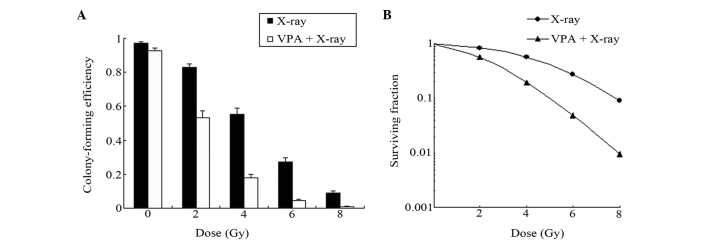
Clonogenic assay at various doses. (A) Results from the clonogenic survival assays indicate that pretreatment of cells with 0.5 mmol/l VPA for 48 h prior to increasing doses (0, 2, 4, 6 and 8 Gy) of radiation results in a decrease in survival compared with irradiation only controls. (B) SERs for combined X-ray and VPA treatment were 2.12 (Dq) and 1.84 (Do), which were 2.76 (Dq) and 2.39 (Do) in the X-ray group. The SER for combined X-ray and VPA treatment, compared with X-ray alone was 1.30. The data are representative of three experiments. Error bars represent one standard deviation. VPA, valproic acid; SER, sensitizer enhancement ratio.

To determine whether VPA altered glioma cell colony formation following irradiation *in vitro*, the effects of VPA on tumor cell radiosensitivity were evaluated. Single cells were seeded in culture conditions and irradiated with a single dose of VPA (0.5 mmol/l) treatment for 48 h. This enhanced radiation-induced cell death and the clonogenic formation at 4, 6 and 8 Gy radiation doses (Fig. 2). VPA enhanced the radiosensitization of the C6 cells in a dose-dependent manner at an SER. The cell SF curve was simulated by a multitarget click mathematical model (Fig. 2), through which the related equation and radioactivity parameters of are obtained. SF is obtained according to the following equation: SF = colony forming numbers of combined group/(numbers of inoculation cells × colony forming numbers of cells without VPA) × 100. The cell survival curve is obtained by taking the irradiation dosage as the abscissa axis and SF as the vertical axis. Do and Dq may be calculated according to the curve. Do represents the average lethal dosage of cells and Dq represents the quasi-field dosage, which indicates the repair ability of cells to sublethal injury. Subsequently the sensitizer enhancement ratio (SER) may be calculated by the following equation: SER = Do value of X-ray group/Do value of combined group The SERs for combined X-ray and VPA treatment were 2.12 (Dq) and 1.84 (Do), which were 2.76 (Dq) and 2.39 (Do) in the X-ray group, respectively. The SER for combined X-ray and VPA treatment, compared to X-ray only, was 1.30.

### VPA increases apoptotic responses to X-ray irradiation by inhibiting Bcl-2 and increasing Bax

Next, the effects of VPA and X-ray exposure on the mRNA and protein expression of substances known to be involved in the apoptotic response were examined. The mRNA and protein levels of Bax and Bcl-2 were determined by PCR and immunoblot analysis.

**Figure 3:**
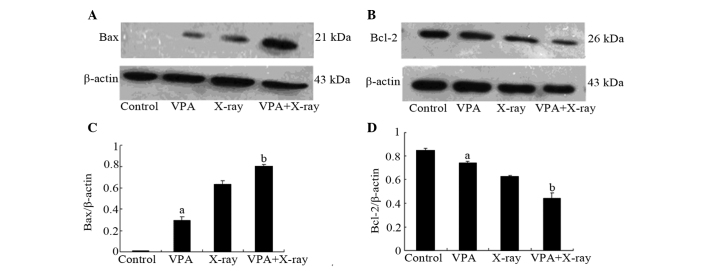
Changes in expression of apoptosis-related proteins following VPA and X-ray. (A and C) VPA and X-ray demonstrated increasing effects on Bax, while the combination group further increased Bax levels. β-actin was included to show equivalent protein loading. (B and D) VPA and X-ray exposure decreased the expression of Bcl-2. The combination of VPA and X-ray further reduced Bcl-2 protein levels. The data are representative of three experiments. Error bars represent one standard deviation. ^a^P<0.05, values were normalized to the control cells; ^b^P<0.05, values were normalized to the control and X-ray-treated cells. VPA, valproic acid.

As shown in Table II and Fig. 3, VPA and X-ray exposure decreased the expression of Bcl-2. The combination of VPA and X-ray further reduced the Bcl-2 mRNA and protein levels. Accordingly, VPA and X-ray demonstrated increasing effects on Bax, while the combination group further increased the Bax levels.

Regardless of the level of transcription or the level of translation, following treatment with VPA, the expression of Bcl-2 mRNA and protein was decreased, whereas upregulation was detected with Bax.

## Discussion

Different HDACIs enhance the radiation response in a variety of malignancies (18), including human brain tumors (19–21), head and neck squamous cell cancer (22), non-small cell lung cancer (23), colorectal cancer (24,25), prostate cancer (9,20), melanoma (26) and metastatic breast cancer (27). However, the mechanisms contributing to HDACI-induced radiosensitization have not been completely identified. Defining the detailed mechanism would aid in the clinical development of HDACIs as radiation sensitizers. The present study further examined the radiosensitizing effects of VPA on the C6 glioma cell line.

The results of this study demonstrated that exposure to VPA leads to a decrease in clonogenic survival and an increase in radiation-induced apoptosis in the rat C6 glioma cell line. VPA also results in modulation of the radiation-induced mitochondrial localization of Bcl-2 and Bax. Taken together, these data indicate an important role of VPA-induced apoptosis in HDACI-mediated radiosensitization.

In fact, histone modification not only enables histone acetyltransferace and histone deacetylase disturbances to lead to tumor development, but it is also able to alter the acetylation status of numerous non-histone targets, including other proteins involved in gene expression, DNA repair, proliferation, migration, cell death, angiogenesis, inflammation and the immune response. These targets are also likely to contribute to tumor progression and resistance to treatments. Accordingly, HDACIs have been shown, in a preclinical setting, to be effective anticancer agents via multiple mechanisms, including the induction of cell cycle arrest, intrinsic and extrinsic apoptotic mechanisms, autophagic cell death, mitotic cell death, the generation of reactive oxygen species, the inhibition of angiogenesis and improvements in NK cell-mediated tumor immunity (6–11,28). A study by Benitez *et al*(29) demonstrated that VPA induces the neuronal-like differentiation of astrocytoma cells. The MTT analysis in the present study showed that VPA, one of the least potent classes of HDACIs (active only at millimolar levels) (16), inhibited the proliferation of rat C6 glioma cells in a time- and dose-dependent manner, which started to become more evident at a concentration of 0.5 mmol/l VPA for 48 h (Table I).

Radiotherapy remains one of the most common forms of cancer treatment, leading to cell death through the induction of DNA double-strand breaks (DSBs and the generation of reactive oxygen species and reactive nitrogen species, which cause non-DNA lesions or extracellular damage, such as lipid perioxidation (30). DNA DSBs generated by radiation may be the most lethal form of damage, although they may be repaired via either homologous recombination or non-homologous end-joining pathways (31,32). However, the lethality of radiation is ascribed to unrepaired or insufficiently repaired DSBs. The Dq-value of survival curves, or the so-called shoulder of survival curves, represents at least in part the cells capability of repairing DNA damage (Fig. 2), although in certain studies there is evidence that cells with a steep survival curve do not necessarily have a recovery deficiency (33).

The exact mechanism of radiosensitization induced by HDACIs was unknown until now. HDACIs were reported to prevent DSB repair and prolong expression of γH2AX, a marker for DNA DSBs, following radiation (34). A variety of HDACIs have been shown to delay the dispersal of radiation-induced γH2AX foci (19,20,23,26,35,36). the present study analyzes the use of VPA in combination with irradiation, focusing on the effects of HDACIs on DNA damage *in vitro*. The SERs for combined treatment, compared with X-ray only, confirm the above mechanism of radiosensitization to a certain extent (Fig. 2). Thus, it appears that VPA prevents DNA DSB repair, which leads to enhanced glioma cell death. DNA damage then occurs as a consequence of the HDACI-enhanced production of irradiation, even at low energy.

Previous studies have demonstrated that the expression of Bax is increased following cell death stimulation, and that Bax then translocates at the membrane of the mitochondria to induce the release of cytochrome *c*(37,38). Additionally, the cleaved form of Bax experiences post-translational modification during apoptosis and is a potent inducer of apoptosis (39,40). Bcl-2 has a role in anti-apoptotic action by inhibiting the pro-apoptotic function of Bax. Cytochrome *c,* which activates caspases, is released from the mitochondria when the proportion of Bax/Bcl-2 increases. Caspases are cysteine proteases that play a key role in cascade activation during apoptosis induced by a number of stimuli (41–44). To investigate the signaling pathways implicated in the radiosensitization-induced apoptosis of the rat C6 cells, an immunoblot analysis was used to examine the Bcl-2 and Bax protein levels, while the mRNA levels were detected by qPCR. Bax expression in the combined group was clearly upregulated relative to the other groups, while Bcl-2 was apparently downregulated at the same time (Table II and Fig. 3). Thus, radiosensitization-induced apoptosis of rat C6 cells occurs, at least to a certain degree, via the Bax/Bcl-2 pathway.

To summarize, VPA in combination with radiotherapy exerts significant antitumor effects *in vitro*. Thus, treatment with VPA in combination with radiotherapy may be a notable approach for the inhibition of the growth of malignant gliomas. Although promising as a sensitizer of radiotherapy, further studies with VPA are warranted. An improved understanding of the mechanisms of VPA-induced radiosensitization are likely to help the development of fresh classes of drugs to treat human diseases and exploit the characteristics of irradiation. Additionally, it is important to investigate other more sensitive HDACIs in combination with radiation to provide drugs that may be better and more suitable for future clinical application.

## Figures and Tables

**Table I:** VPA reduces survival of C6 cells in a dose- and time-dependent manner.

VPA, mmol/l	Cell suppression ratio, %		

	24 h	48 h	72 h
0.25	4.139±1.523^a^	5.399±2.010^a^	7.018±3.314^a^
0.5	11.479±1.306^a^	19.963±1.164^a^	25.168±2.664^a^
1	14.197±1.334^a^	24.588±1.688^a^	34.415±3.945^a^
2	19.062±0.812^a^	29.448±2.064^a^	39.859±2.964^a^
4	27.355±0.345^a^	38.244±3.165^a^	50.353±1.650^a^

a P<0.05, values normalized to the control cells.

VPA, valproic acid.

**Table II:** mRNA expression of Bcl-2 and Bax.

mRNA	Control	VPA	X-ray	VPA + X-ray
Bcl-2	0.816±0.051^a^	0.652±0.055^a^	0.508±0.046^a^	0.247±0.041^b^
Bax	0.236±0.045^a^	0.339±0.025^a^	0.528±0.049^a^	0.856±0.034^b^

a P<0.01, values normalized to the control cells;

b P<0.01, values normalized to the control and X-ray-treated cells.

VPA, valproic acid.
